# Association between fasting stress hyperglycemia ratio and contrast-induced acute kidney injury in coronary angiography patients: a cross-sectional study

**DOI:** 10.3389/fendo.2023.1300373

**Published:** 2023-12-13

**Authors:** Yu Shan, Maoning Lin, Fangfang Gu, Shuxin Ying, Xiaoyi Bao, Qiongjun Zhu, Yecheng Tao, Zhezhe Chen, Duanbin Li, Wenbin Zhang, Guosheng Fu, Min Wang

**Affiliations:** ^1^ Department of Cardiology, Sir Run Run Shaw Hospital, College of Medicine, Zhejiang University, Hangzhou, Zhejiang, China; ^2^ Key Laboratory of Cardiovascular Intervention and Regenerative Medicine of Zhejiang Province, Hangzhou, China; ^3^ Department of Cardiology, The Affiliated Huzhou Hospital (Huzhou Central Hospital), College of Medicine, Zhejiang University, Huzhou, Zhejiang, China; ^4^ Department of Endocrinology and Metabolism, Sir Run Run Shaw Hospital, College of Medicine, Zhejiang University, Hangzhou, Zhejiang, China

**Keywords:** fasting stress hyperglycemia ratio, contrast-induced acute kidney injury, serum creatinine elevation, coronary angiography, diabetes

## Abstract

**Aims:**

Stress hyperglycemia ratio (SHR), an emerging indicator of critical illness, exhibits a significant association with adverse cardiovascular outcomes. The primary aim of this research endeavor is to evaluate the association between fasting SHR and contrast-induced acute kidney injury (CI-AKI).

**Methods:**

This cross-sectional study comprised 3,137 patients who underwent coronary angiography (CAG) or percutaneous coronary intervention (PCI). The calculation of fasting SHR involved dividing the admission fasting blood glucose by the estimated mean glucose obtained from glycosylated hemoglobin. CI-AKI was assessed based on elevated serum creatinine (Scr) levels. To investigate the relationship between fasting SHR and the proportion of SCr elevation, piecewise linear regression analysis was conducted. Modified Poisson’s regression analysis was implemented to evaluate the correlation between fasting SHR and CI-AKI. Subgroup analysis and sensitivity analysis were conducted to explore result stability.

**Results:**

Among the total population, 482 (15.4%) patients experienced CI-AKI. Piecewise linear regression analysis revealed significant associations between the proportion of SCr elevation and fasting SHR on both sides (≤ 0.8 and > 0.8) [β = -12.651, 95% CI (−23.281 to −2.022), P = 0.020; β = 8.274, 95% CI (4.176 to 12.372), P < 0.001]. The Modified Poisson’s regression analysis demonstrated a statistically significant correlation between both the lowest and highest levels of fasting SHR and an increased incidence of CI-AKI [(SHR < 0.7 vs. 0.7 ≤ SHR < 0.9) β = 1.828, 95% CI (1.345 to 2.486), P < 0.001; (SHR ≥ 1.3 vs. 0.7 ≤ SHR < 0.9) β = 2.896, 95% CI (2.087 to 4.019), P < 0.001], which was further validated through subgroup and sensitivity analyses.

**Conclusion:**

In populations undergoing CAG or PCI, both lowest and highest levels of fasting SHR were significantly associated with an increased occurrence of CI-AKI.

## Introduction

Coronary artery disease (CAD) imposes a significant global disease burden and stands as a prominent contributor to human mortality on a global scale (1, 2). Recently, with the widespread application of interventional techniques such as coronary angiography (CAG) and percutaneous coronary intervention (PCI) in the diagnosis and treatment of CAD, there has been a significant improvement in the clinical prognosis of patients with CAD (3–6). However, accompanying this advancement are a series of related complications that cannot be overlooked due to their impact on patient health (7, 8).

CAG and PCI critically depend on the utilization of iodinated intravascular contrast agents to facilitate vessel and chamber imaging. However, it should be noted that contrast agents utilized in CAG and PCI procedures may give rise to a complication known as contrast-induced acute kidney injury (CI-AKI) (9, 10). This condition is characterized by the acute impairment of renal function caused by the administration of iodinated contrast agents and is currently considered the third principal contributor to iatrogenic renal dysfunction (11). Defined by the European Society of Urogenital Radiology (ESUR), CI-AKI is characterized as an elevation in serum creatinine (Scr) levels exceeding 44.2 µmol/L (0.5 mg/dL) or 25% from baseline within 72 hours post-administration of contrast media (12). The incidence of CI-AKI can vary depending on the diagnostic criteria, study population, and preventive measures employed. Current research has shown that risk factors such as diabetes, anemia, baseline renal insufficiency, severity of heart failure, contrast agent dosage, and type of contrast agent are associated with the occurrence of CI-AKI (13–15). Given that CI-AKI can prolong hospital stay, increase healthcare costs, and potentially lead to more severe complications, early identification of these risk factors is of paramount importance in the timely identification and mitigation of CI-AKI.

Pre-procedural glucose levels have garnered recognition as a risk factor contributing to CI-AKI (14). However, relying solely on blood glucose levels may not fully reflect the acute hyperglycemic state, as they could also be influenced by chronic glucose levels. Recently, one study has reported a novel biomarker, the stress hyperglycemia ratio (SHR), which is considered a superior prognostic indicator for critical illness compared to absolute hyperglycemia (16). SHR is calculated based on admission blood glucose (ABG) and glycosylated hemoglobin A1c (HbA1c) levels (16). In contrast to ABG levels, SHR has the ability to quantify the extent of the relative increase in glycemic levels from chronic glycemia over a period of 8 to 12 weeks. Current research has demonstrated that SHR can serve as a significant predictor for cardiovascular and cerebrovascular diseases, as well as respiratory tract infections (17–20). Moreover, meal timing is associated with blood glucose levels, and considering that some patients may have already eaten when experiencing ischemic symptoms while others may experience symptoms before meals, utilizing fasting blood glucose (FBG) to calculate fasting SHR may provide a more accurate reflection of the extent of acute stress-induced hyperglycemia, with previous research reporting its predictive value for cardiovascular diseases (21, 22). Nevertheless, the correlation between CI-AKI and fasting SHR remains unclear. Therefore, the primary focus of this study encompassed an examination of the association between fasting SHR and the incidence of CI-AKI among individuals subjected to CAG or PCI.

## Methods

### Study population

At Sir Run Run Shaw Hospital and its consortium of affiliated medical hospitals, this retrospective cross-sectional cohort study was employed. The study process strictly adhered to the principles outlined in the Declaration of Helsinki, ensuring rigorous ethical standards were maintained throughout. Prior to the initiation of the study, the independent Ethics Committee of Sir Run Run Shaw Hospital granted thorough approval for this study under the reference number 20220228-30, validating the ethical integrity of the research process.

**Figure 1:**
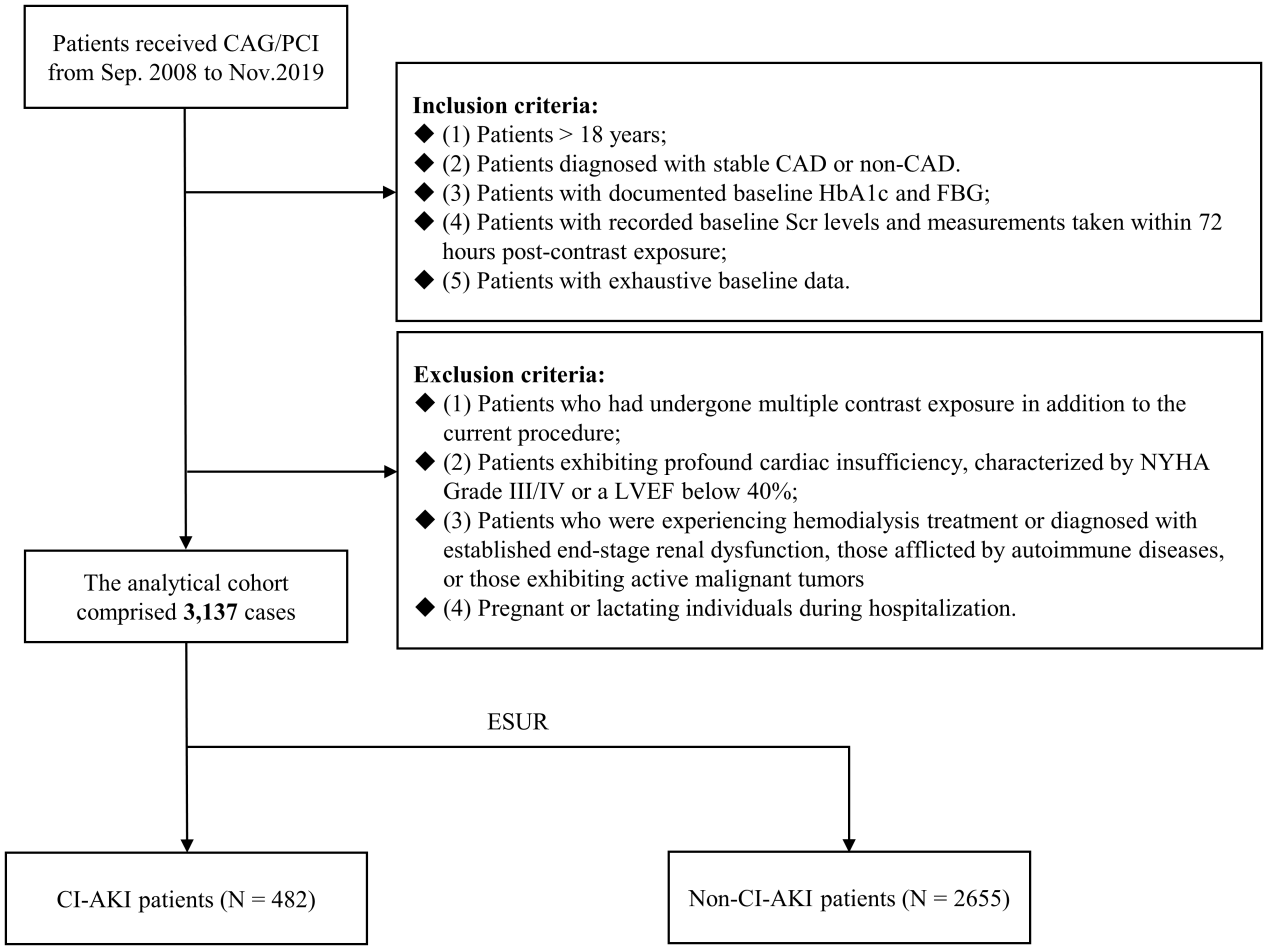
Flowchart of patient enrollment. CAG, coronary angiography; PCI, percutaneous coronary intervention; CAD, coronary artery disease; HbA1c, glycosylated hemoglobin A1c; FBG, fasting blood glucose; Scr, serum creatinine; NYHA, New York Heart Association; LVEF, left ventricular ejection fraction; CI-AKI, contrast-induced acute kidney injury.

**Figure 1** illustrates the flowchart depicting the process of patient screening. From September 2008 to November 2019, a cumulative number of 3137 patients experienced CAG or PCI and met the following diagnostic criteria: (1) patients > 18 years; (2) patients diagnosed with stable CAD or non-CAD. (3) patients with documented baseline HbA1c and FBG; (4) patients with recorded baseline Scr levels and measurements taken within 72 hours post-contrast exposure; (5) patients with exhaustive baseline data. The exclusion criteria comprised the following: (1) In addition to the current procedure, patients had undergone multiple exposure to contrast agents; (2) patients exhibiting profound cardiac insufficiency, characterized by New York Heart Association Grade III/IV or a left ventricular ejection fraction (LVEF) below 40%; (3) patients who were experiencing hemodialysis treatment or diagnosed with established end-stage renal dysfunction, those afflicted by autoimmune diseases, or those exhibiting active malignant tumors; and (4) pregnant or lactating individuals during hospitalization.

### Data collection

Upon hospital admission, baseline characteristics including age, sex, LVEF, average heart rate, recent medication usage, and past medical conditions were queried and recorded by trained research coordinators from participating hospitals. Detailed documentation of the specific features pertaining to CAG or PCI was meticulously performed postoperatively. Within a 24-hour period following hospital admission, venous blood samples for the assessment of HbA1c levels and other laboratory parameters were meticulously obtained from the cubital vein. Additionally, following a minimum fasting period of 8 hours, FBG levels were measured through the collection of venous blood samples following an overnight fast. The baseline Scr concentration was determined through biochemical testing upon admission. Subsequently, following exposure to the contrast medium, postoperative Scr concentration was measured on a minimum of three occasions within a 72-hour period, with the highest recorded value being documented.

### Definitions

The average glucose levels were estimated from HbA1c, employing the subsequent formula: estimated average glucose level (mmol/L) = 1.59 x HbA1c (%) – 2.59 (23). Fasting SHR is calculated as FBG at admission (mmol/L)/estimated average glucose level (mmol/L) (16). The fasting SHR was partitioned into two distinct segments based on a threshold of 0.8 (SHR ≤ 0.8 and SHR > 0.8). Furthermore, a meticulous categorization strategy was employed to further stratify fasting SHR into five discrete groups, each defined by equal intervals of 0.2 (cut-off points: < 0.7, 0.7-0.9, 0.9-1.1, 1.1-1.3, ≥ 1.3).

As per the guidelines established by ESUR, CI-AKI is defined as a rise in Scr level exceeding 44.2 µmol/L (0.5 mg/dl) or a 25% increment from the individual’s baseline value, occurring within a timeframe of 72 hours subsequent to the administration of contrast agents, and other causes of renal insufficiency should also be excluded (12).

### Statistical analysis

Categorical variables were reported as counts with corresponding percentages, and their comparisons were performed using either the Chi-square test or Fisher’s exact test. Continuous variables were represented by their mean value accompanied by the standard deviation (SD), and if they followed a normal distribution, Student’s t-test was utilized for evaluation. Conversely, for continuous variables that did not exhibit a normal distribution, their description included the median value coupled with the interquartile range, and they were subjected to analysis utilizing the Mann-Whitney U-test.

The restricted cubic spline (RCS) model was utilized to visually represent the correlation between fasting SHR and CI-AKI. It is apparent that the relative risk of CI-AKI significantly increases with both high and low fasting SHR values when fasting SHR around 0.8 was used as the reference point. Therefore, fasting SHR was divided into two segments based on a threshold value of 0.8 for conducting piecewise linear regression analysis with the aim of investigating the linearity trend on both ends of fasting SHR (SHR ≤ 0.8 and SHR > 0.8). By incorporating age, gender (male or female), estimated glomerular filtration rate (eGFR), hypertension, type of contrast agent (isotonic or hypotonic), volume of contrast, LVEF, C-reactive protein (CRP) (< 5, 5-10, or ≥ 10mg/L), and medications (administration of statin, furosemide injection and dopamine) (yes or no) as covariates, the multivariable regression analysis effectively controlled for potential significant predictors, with these included factors having been substantiated by previous research (24). Subsequently, employing equal interval categorization, the fasting SHR was divided into five groups, and a modified Poisson’s regression analysis was conducted to determine the adjusted relative risk (aRR) of fasting SHR for the occurrence of CI-AKI. Similar covariates as mentioned above were utilized for adjusting the aRR as well. Moreover, a comprehensive exploratory analysis was performed by stratifying the population into subgroups based on diabetes (yes or no) and HbA1c levels (≤ 6.0% or > 6.0%). This subgroup analysis was conducted using a modified Poisson’s regression model with same covariates mentioned above. In the end, to assess the soundness of the result, two sensitivity analyses were conducted, involving the exclusion of patients who experienced hypoglycemia throughout the initial assessment period and those who encountered a hyperglycemic crisis at the beginning of the study.

A statistically significant result was established based on a two-tailed P-value < 0.05. The manipulation and evaluation of data were conducted utilizing the SPSS software, specifically version 23.0 developed by IBM, with its origin in Chicago, Illinois. Additionally, the analysis also involved the utilization of R version 4.1.2 (developed in Vienna, Austria).

## Results

### Baseline characteristics

Within the study, a cohort comprising 3137 individuals who experienced either CAG or PCI was included. **Table 1** provides an overview of the primary characteristics relating to baseline demographics and clinical variables.

**Table 1:** Baseline characteristics of the two groups.

		CI-AKI		*P* value
	Overall (n=3137)	No (n=2655)	Yes (n=482)	
Demographic features				
Age, yrs	66.92 ± 10.81	66.44 ± 10.67	69.56 ± 11.21	<0.001*
Male, n (%)	2100 (66.9)	1814 (68.3)	286 (59.3)	<0.001*
Hypertension, n (%)	2055 (65.5)	1717 (64.7)	338 (70.1)	0.020*
Diabetes, n (%)	863 (27.5)	692 (26.1)	171 (35.5)	<0.001*
LVEF, %	59.33 ± 13.31	60.07 ± 13.12	55.34 ± 13.62	<0.001*
Average heart rate, beats/min	73.82 ± 38.40	73.56 ± 40.96	75.26 ± 18.73	0.370
Laboratory information				
Fasting SHR	0.92 ± 0.30	0.91 ± 0.27	1.01 ± 0.40	<0.001*
FBG, mmol/L	7.01 ± 3.13	6.83 ± 2.82	8.03 ± 4.32	<0.001*
HbA1c, %	6.45 ± 1.39	6.41 ± 1.33	6.68 ± 1.65	<0.001*
Serum creatinine at baseline, μmol/L	77.0 [65.0, 95.0]	77.0 [65.0, 94.0]	74.0 [60.8, 103.3]	0.191
Serum creatinine elevation, %	3.8 [-4.7, 16.0]	1.3 [-6.3, 9.1]	39.7 [31.0, 54.3]	<0.001*
eGFR, ml/(min×1.73 m^2^)	78.53 ± 23.24	79.14 ± 22.08	75.17 ± 28.61	0.001*
Hemoglobin, g/L	129.82 ± 19.59	131.33 ± 18.77	121.62 ± 21.79	<0.001*
High density lipoprotein, mmol/L	1.02 ± 0.28	1.03 ± 0.28	0.99 ± 0.30	0.007*
White blood cell, ×10^9^/L	7.17 ± 2.76	6.96 ± 2.52	8.31 ± 3.64	<0.001*
C-reactive protein, mg/L	2.20 [0.90, 7.05]	1.90 [0.80, 6.30]	4.20 [1.40, 15.95]	<0.001*
Procedure data				
Only CAG procedure	1876 (59.8)	1599 (60.2)	277 (57.5)	0.267
PCI procedure	1261 (40.2)	1056 (39.8)	205 (42.5)	0.267
Types of contrast agent, n (%)				
Isotonic	1103 (35.2)	933 (35.1)	170 (35.3)	0.957
Hypotonic	2034 (64.8)	1722 (64.9)	312 (64.7)	0.957
Volume of contrast agent, mg	80.0 [50.0, 150.0]	80.0 [50.0, 140.0]	100.0 [60.0, 150.0]	0.001*
CTO, n (%)	155 (4.9)	135 (5.1)	20 (4.1)	0.383
Total length of stents, mm	40.0 [27.5, 66.0]	42.0 [27.5, 66.0]	38.0 [27.3, 60.0]	0.472
Medication, n (%)				
ACEI	536 (17.1)	440 (16.6)	96 (19.9)	0.073
ARB	1040 (33.2)	895 (33.7)	145 (30.1)	0.120
CCB	925 (29.5)	784 (29.5)	141 (29.3)	0.903
Statin	2602 (82.9)	2214 (83.4)	388 (80.5)	0.120
Dopamine	912 (29.1)	704 (26.5)	208 (43.2)	<0.001*
Furosemide injection	483 (15.4)	331 (12.5)	152 (31.5)	<0.001*

Categorical variables were reported as counts with corresponding percentages and continuous variables are reported as either the mean ± standard deviation or the median (interquartile range). CI-AKI, contrast-induced acute kidney injury; LVEF, left ventricular ejection fraction; SHR, stress hyperglycemia ratio; FBG, fasting blood glucose; HbA1c: glycosylated hemoglobin A1c; eGFR, estimated glomerular filtration rate; CAG, coronary angiography; PCI, percutaneous coronary intervention; CTO, chronic total occlusion; ACEI, angiotensin converting enzyme inhibitor; ARB, angiotensin receptor blocker; CCB, calcium channel blocker; *P < 0.05.

The study cohort had an average age of 66.92 ± 10.81 years, with males representing 66.9% of the participants. Among this population, a notable subset of 482 subjects experienced CI-AKI following CAG or PCI. In the cohort of patients with CI-AKI, there was a higher prevalence of hypertension (70.1% vs. 64.7%, P = 0.020) and diabetes (35.5% vs. 26.1%, P < 0.001), accompanied by a lower proportion of individuals receiving furosemide injection (12.5% vs. 31.5%, P < 0.001) and dopamine (26.5% vs. 43.2%, P < 0.001). Furthermore, these patients exhibited lower levels of hemoglobin (121.62 ± 21.79 g/L vs. 131.33 ± 18.77g/L, P < 0.001), baseline LVEF (55.34 ± 13.62% vs. 60.07 ± 13.12%, P < 0.001), and baseline eGFR, along with higher levels of fasting SHR (1.01 ± 0.40 vs. 0.91 ± 0.27, P < 0.001), FBG (8.03 ± 4.32mmol/L vs. 6.83 ± 2.82 mmol/L, P < 0.001), and white blood cell (8.31 ± 3.64×109/L vs. 6.96 ± 2.52×109/L, P < 0.001). Nevertheless, no significant distinctions were observed regarding the average heart rate or the proportions of patients using calcium channel blocker (CCB), angiotensin converting enzyme inhibitor (ACEI), angiotensin receptor blocker (ARB), and statin between the two groups.

### Association between fasting SHR and proportion of Scr elevation

The study presents the results of the multivariable piecewise linear regression analysis (**Table 2**), which included calculation of the β coefficient and its 95% confidence interval (CI), for both sides of fasting SHR (SHR ≤ 0.8 and SHR > 0.8). After comprehensive adjustment, the analysis revealed a significantly negative correlation between fasting SHR and the proportion of SCr elevation when fasting SHR ≤ 0.8 [β = -12.651, 95% CI (−23.281 to −2.022), P = 0.020]. Conversely, a significant positive relationship emerged when the fasting SHR was > 0.8 [β = 8.274, 95% CI (4.176 to 12.372), P < 0.001].

**Table 2:** Piecewise linear regression analysis of fasting SHR with the proportion of SCr elevation.

Fasting SHR	Cases/Overall (%)	Model 1		Model 2		Model 3	
		β (95%CI)	*P* value	β (95%CI)	*P* value	β (95%CI)	*P* value
≤ 0.8	167/1260 (13.3%)	-18.625[-29.512 to -7.737]	0.001*	-16.794[-27.724 to -5.865]	0.003*	-12.651[-23.281 to -2.022]	0.020*
> 0.8	315/1877 (16.8%)	12.039[7.877 to 16.201]	<0.001*	10.023[5.859 to 14.188]	<0.001*	8.274[4.176 to 12.372]	<0.001*

Model 1: Adjusted for none.

Model 2: Adjusted for age, gender (male or female), hypertension (yes or no), eGFR, type of contrast agent (isotonic or hypotonic), volume of contrast, and LVEF.

Model 3: Additionally adjusted for CRP (<5, 5-10, or ≥10mg/L), and medications (administration of statin, furosemide injection and dopamine) (yes or no).

SHR, stress hyperglycemia ratio; Scr, serum creatinine; CI, confidence interval; eGFR, estimated glomerular filtration rate; LVEF, left ventricular ejection fraction; CRP, C-reactive protein; *P < 0.05.

### The association of the fasting SHR with CI−AKI

**Figure 2:**
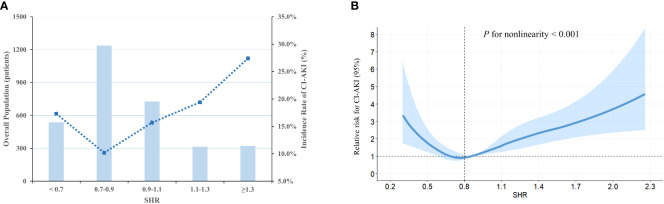
The population distribution histogram and RCS curve. **(A)** Distribution of population and the incidence of CI-AKI based on fasting SHR categories. The changing trend in the incidence of CI-AKI is illustrated by the blue dashed line. The bar plots presented the distribution of the population across different categories of fasting SHR. Left axis, population count (patients); right axis, incidence of CI-AKI (%). **(B)** RCS analysis is employed to investigate the non-linear relationship between fasting SHR and CI-AKI. The relative risk of fasting SHR for CI-AKI is represented by the solid blue line, while the shaded area surrounding the line represents the 95% CI of the curve. SHR, stress hyperglycemia ratio; CI-AKI, contrast-induced acute kidney injury; RCS, restricted cubic spline; CI, confidence interval.

The study cohort underwent categorization into five groups, employing equally spaced intervals of the fasting SHR value (Predefined cut-off points: < 0.7, 0.7-0.9, 0.9-1.1, 1.1-1.3, ≥ 1.3). Based on the graphical representation depicted in **Figure 2**, the group exhibiting a fasting SHR value ranging from 0.7 to 0.9 demonstrates the lowest occurrence of CI-AKI, with an increasing occurrence of CI-AKI as the fasting SHR value deviates in either direction. Additionally, **Figure 2** demonstrates a statistically significant J-shaped non-linear relationship between fasting SHR and CI-AKI, as ascertained utilizing the RCS modeling approach (P-value indicating non-linearity < 0.001), even after adjusting for other confounding factors. Notably, a significant reduction in the relative risk of CI-AKI was observed when the fasting SHR was approximately 0.8.

Subsequently, a modified Poisson’s regression analysis was utilized to assess and model the relationship between fasting SHR and CI-AKI, with the second category (0.7 ≤ SHR < 0.9) serving as the reference group (refer to **Table 3** for detailed results). After conducting multivariable adjustment for potential confounders and employing multiple model analyses, a significant association was observed between the low level of fasting SHR and the incidence of CI-AKI [(SHR < 0.7 vs. 0.7 ≤ SHR < 0.9) β = 1.828, 95% CI (1.345 to 2.486), P < 0.001]. Furthermore, there was also a significant association between the higher level of fasting SHR and the occurrence rate of CI-AKI [(0.9 ≤ SHR < 1.1 vs. 0.7 ≤ SHR < 0.9) β = 1.623, 95% CI (1.220 to 2.158), P = 0.001; (1.1 ≤ SHR < 1.3 vs. 0.7 ≤ SHR < 0.9) β = 2.082, 95% CI (1.456 to 2.975), P < 0.001; (SHR ≥ 1.3 vs. 0.7 ≤ SHR < 0.9) β = 2.896, 95% CI (2.087 to 4.019), P < 0.001].

**Table 3:** Modified Poisson’s regression analysis of fasting SHR categories with CI-AKI.

Fasting SHR	Cases/Overall (%)	Model 1		Model 2		Model 3	
		aRR (95%CI)	*P* value	aRR (95%CI)	*P* value	aRR (95%CI)	*P* value
< 0.7	93/538 (17.3%)	1.843 [1.379 to 2.462]	< 0.001*	1.834 [1.359 to 2.477]	< 0.001*	1.828 [1.345 to 2.486]	< 0.001*
≤ 0.7, < 0.9	126/1237 (10.2%)	1 (Reference)		1 (Reference)		1 (Reference)	
≤ 0.9, < 1.1	114/727 (15.7%)	1.640 [1.249 to 2.152]	< 0.001*	1.618 [1.224 to 2.139]	0.001*	1.623 [1.220 to 2.158]	0.001*
≤ 1.1, < 1.3	61/314 (19.4%)	2.126 [1.521 to 2.971]	< 0.001*	2.133 [1.506 to 3.019]	< 0.001*	2.082 [1.456 to 2.975]	< 0.001*
≥ 1.3	88/321 (27.4%)	3.330 [2.451 to 4.526]	< 0.001*	3.179 [2.314 to 4.367]	< 0.001*	2.896 [2.087 to 4.019]	< 0.001*
*P* for trend			< 0.001*		< 0.001*		< 0.001*

Model 1: Adjusted for none.

Model 2: Adjusted for age, gender (male or female), hypertension (yes or no), eGFR, type of contrast agent (isotonic or hypotonic), volume of contrast, and LVEF.

Model 3: Additionally adjusted for CRP (<5, 5-10, or ≥10mg/L), and medications (administration of statin, furosemide injection and dopamine) (yes or no).

SHR, stress hyperglycemia ratio; CI-AKI, contrast-induced acute kidney injury; aRR, adjusted relative risk; CI, confidence interval; eGFR, estimated glomerular filtration rate; LVEF, left ventricular ejection fraction; CRP, C-reactive protein; *P < 0.05.

### Subgroup analysis and sensitivity analysis

**Figure 3:**
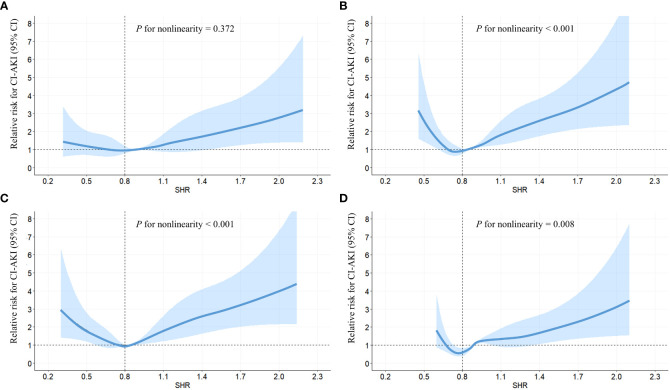
RCS analyses between fasting SHR and CI-AKI in different subgroups. RCS analyses were performed by stratifying the population into subgroups based on diabetes (yes **(A)** or no **(B)**) and HbA1c levels (> 6.0% **(C)** or ≤ 6.0% **(D)**); The relative risk of fasting SHR for CI-AKI is represented by the solid blue line, while the shaded area surrounding the line represents the 95% CI of the curve. CI-AKI, contrast-induced acute kidney injury; CI, confidence interval; RCS, restricted cubic spline; SHR, stress hyperglycemia ratio.

Following adjustment for confounding factors, an exploration was undertaken to ascertain the robustness of the correlation between fasting SHR and the occurrence of CI-AKI by stratifying individuals according to the presence of diabetes and HbA1c levels (**Tables 4**, **5**). Subgroup analyses demonstrated a strong association between both the lowest and highest levels of fasting SHR and a higher rate of CI-AKI regardless of whether patients had comorbid diabetes or not. Moreover, in both the HbA1c ≤ 6% and HbA1c > 6% subgroups, a significant correlation between the lowest and highest levels of fasting SHR and the incidence of CI-AKI was also identified. These findings were consistent with the primary outcomes observed within the overall population. Within **Figure 3**, the application of the RCS model visually represents the association between fasting SHR and CI-AKI across different subgroups.

**Table 4:** Subgroup analysis according to stratification of diabetes.

Subgroups	Fasting SHR	Cases/overall (%)	aRR (95% CI)	*P* value
No diabetes	< 0.7	44/314 (14.0%)	1.680 (1.116 to 2.529)	0.013*
311/2274 (13.7%)	≤ 0.7, < 0.9	94/1010 (9.3%)	1 (Reference)	
	≤ 0.9, < 1.1	80/543 (14.7%)	1.711 (1.225 to 2.391)	0.002*
	≤ 1.1, < 1.3	41/212 (19.3%)	2.316 (1.509 to 3.556)	< 0.001*
	≥ 1.3	52/195 (26.7%)	2.984 (1.980 to 4.497)	< 0.001*
Diabetes	< 0.7	49/224 (21.9%)	1.778 (1.048 to 3.014)	0.033*
171/863 (19.8%)	≤ 0.7, < 0.9	32/227 (14.1%)	1 (Reference)	
	≤ 0.9, < 1.1	34/184 (18.5%)	1.455 (0.827 to 2.560)	0.193
	≤ 1.1, < 1.3	20/102 (19.6%)	1.602 (0.822 to 3.125)	0.167
	≥ 1.3	36/126 (28.6%)	2.599 (1.455 to 4.641)	0.001*

Adjusted for age, gender (male or female), hypertension (yes or no), eGFR, type of contrast agent (isotonic or hypotonic), volume of contrast, LVEF, CRP (<5, 5-10, or ≥10mg/L), and medications (administration of statin, furosemide injection and dopamine) (yes or no).

SHR, stress hyperglycemia ratio; aRR, adjusted relative risk; CI, confidence interval; eGFR, estimated glomerular filtration rate; LVEF, left ventricular ejection fraction; CRP, C-reactive protein; *P < 0.05.

**Table 5:** Subgroup analysis according to stratification of HbA1c levels.

Subgroups	Fasting SHR	Cases/overall (%)	aRR (95% CI)	*P* value
HbA1c ≤ 6%	< 0.7	20/123 (16.3%)	2.045 (1.112 to 3.759)	0.021*
221/1591 (13.9%)	≤ 0.7, < 0.9	59/690 (8.6%)	1 (Reference)	
	≤ 0.9, < 1.1	70/450 (15.6%)	2.055 (1.392 to 3.034)	< 0.001*
	≤ 1.1, < 1.3	34/180 (18.9%)	2.381 (1.451 to 3.908)	0.001*
	≥ 1.3	38/148 (25.7%)	2.725 (1.655 to 4.486)	< 0.001*
HbA1c > 6%	< 0.7	73/415 (17.6%)	1.605 (1.100 to 2.342)	0.014*
261/1546 (16.9%)	≤ 0.7, < 0.9	67/547 (12.2%)	1 (Reference)	
	≤ 0.9, < 1.1	44/277 (15.9%)	1.222 (0.791 to 1.888)	0.366
	≤ 1.1, < 1.3	27/134 (20.1%)	1.876 (1.107 to 3.181)	0.019*
	≥ 1.3	50/173 (28.9%)	2.911 (1.863 to 4.549)	< 0.001*

Adjusted for age, gender (male or female), hypertension (yes or no), eGFR, type of contrast agent (isotonic or hypotonic), volume of contrast, LVEF, CRP (<5, 5-10, or ≥10mg/L), and medications (administration of statin, furosemide injection and dopamine) (yes or no).

HbA1c, glycosylated hemoglobin A1c; SHR, stress hyperglycemia ratio; aRR, adjusted relative risk; CI, confidence interval; eGFR, estimated glomerular filtration rate; LVEF, left ventricular ejection fraction; CRP, C-reactive protein; *P < 0.05.

To assess the soundness of the study outcomes, two sensitivity analyses were conducted, including the exclusion of patients who experienced hypoglycemia throughout the initial assessment period and those who encountered a hyperglycemic crisis at the beginning of the study. Consistently, in both sensitivity analyses, a strong and statistically significant correlation was identified between the uppermost and lowermost ranges of fasting SHR and an elevated incidence of CI-AKI (**Supplementary Tables 1**, **2**). Furthermore, the RCS modeling visually portrays the non-linear correlation between fasting SHR and CI-AKI in both sensitivity analyses (**Supplementary Figure 1**), aligning consistently with the findings observed in the overall population.

## Discussion

The study demonstrated that within the cohort of individuals undergoing CAG or PCI, both the lowest and highest levels of fasting SHR exhibited a significant correlation with an increased incidence of CI-AKI. Importantly, this correlation was observed irrespective of whether the patients had diabetes or HbA1c > 6%.

Diabetes and preoperative blood glucose levels are now recognized as independent risk factors for CI-AKI (14, 24). However, previous studies have suggested that daily glycemic fluctuations (glycemic variability) may pose a greater risk for adverse cardiovascular events compared to sustained hyperglycemia (25, 26). Therefore, the glycemic variability is an important aspect that requires close attention. The ABG level alone cannot fully reflect the acute elevation of glucose upon hospital admission, as it is also influenced by the long-term glucose, especially in poorly controlled diabetic patients. Several studies have suggested that SHR may be a reliable biomarker for critical illness (16, 27). SHR is determined through the division of the ABG by the average glucose level obtained from HbA1c, which theoretically quantifies the relative glycemic rise compared to chronic glycemia over the past 8-12 weeks. Given the extensive utilization of glucose and HbA1c testing in clinical practice, coupled with the straightforward nature of SHR calculation, it is evident that SHR can be employed as a valuable tool for delivering prognostic information to hospitalized patients.

Several studies have already demonstrated the correlation between SHR and major adverse cardiovascular and cerebrovascular events (MACCE) (28–31). Yang reported that a high level of SHR might be an effective predictor for the incidence of MACCE after PCI (31). However, it is worth noting that this study might have overlooked the potential impact of low SHR levels on the incidence of MACCE. Additionally, the study did not consider the possibility of a non-linear relationship between SHR and the incidence of MACCE. According to Robert’s finding (16), the SHR has the potential to be a superior biomarker in predicting critical illnesses as compared to absolute high blood glucose levels. However, it is plausible that this study might neglect the potential correlation between the SHR calculated at lower blood glucose levels and patient prognosis. the SHR might potentially serve as a superior biomarker compared to absolute high blood glucose, but this study might disregard the relationship between SHR at lower blood glucose concentrations and patient prognosis. Therefore, in contrast to previous research that primarily categorized SHR based on quartiles or quintiles (27, 29, 32, 33), this extensive-scale study not only implemented more meticulous and specific categorizations but also accounted for the non-linear association between CI-AKI and SHR, as well as the influence of lower levels of SHR on CI-AKI. Additionally, meal timing also exerts an influence on ABG. In clinical practice, it is observed that certain patients may have already eaten when experiencing ischemic symptoms, while others may manifest symptoms prior to meals. Consequently, we postulated that the calculation of fasting SHR using FBG and HbA1c can more accurately reflect the degree of acute stress-induced hyperglycemia. According to our research findings, it is recommended that physicians pay more attention to patients with lower or higher fasting SHR values and take measures to prevent the occurrence of CI-AKI. For instance, in the case of diabetic patients, it is advisable to use a rational hypoglycemic regimen, avoiding excessive intensification of hypoglycemic medications that may cause a significant decrease in blood glucose levels. Additionally, informing preoperative patients about the importance of adhering to a balanced diet and avoiding excessive food consumption can prevent drastic fluctuations in blood glucose levels. Lastly, enhancing perioperative blood glucose monitoring can assist physicians in better understanding the dynamic changes in patients’ blood glucose levels. Furthermore, as shown in **Table 1**, a lower proportion of furosemide injection was observed in our study, which may be attributed to the fact that furosemide injection was not included in routine hydration therapy at our study center. Therefore, furosemide injection was only administered to patients with relevant comorbidities such as oedema and hypertension.

The association between high level fasting SHR and CI-AKI can be elucidated through several mechanisms. Firstly, stress hyperglycemia may engender increased stress responses, encompassing mitochondrial dysfunction, oxidative stress, endoplasmic reticulum stress, thereby collectively precipitating a cascade of pathological alterations within renal tissue, ultimately culminating in renal dysfunction and an escalated susceptibility to CI-AKI (34–39). Secondly, the equilibrium between nitric oxide (NO) bioavailability and the accumulation of reactive oxygen species (ROS) in endothelial cells may be disrupted by stress hyperglycemia, which may cause endothelial dysfunction and subsequently induce CI-AKI (40–42). Additionally, stress hyperglycemia may also stimulate the advanced glycation end products (AGEs), leading to microvascular damage and subsequent renal glomerulosclerosis, which may further increase the occurrence of CI-AKI (43, 44). The mechanisms underlying how low levels of fasting SHR contribute to CI-AKI are still unclear. Nevertheless, it remains within the realm of reasoned speculation to provide plausible explanations. From the calculation formula of fasting SHR, patients with low fasting SHR values are often associated with lower blood glucose levels. Previous studies have reported that under hypoglycemic conditions, sympathetic adrenal activation leads to increased levels of adrenaline and noradrenaline, which subsequently cause hemodynamic changes. Additionally, it can also induce endothelial dysfunction, elevate inflammation levels, and increase levels of tissue plasminogen activator and aldosterone. These factors collectively increase the risk of systemic atherosclerosis, including renal artery sclerosis, which may partly explain the association between low fasting SHR levels and increased incidence of CI-AKI (45–47). Furthermore, some patients with low fasting SHR values may also exhibit higher levels of HbA1c. This may indicate poor glycemic control in these patients during routine times, but hypoglycemic episodes occur before CAG/PCI procedure. Such patients display significant glycemic fluctuations, which previous studies have shown could impair vascular endothelial function, increase inflammation levels, and subsequently cause renal artery injury leading to CI-AKI (48, 49).

There are several limitations to this study that should be acknowledged. Firstly, the calculation of fasting SHR requires patients to obtain blood glucose values in a fasting state, which may not be practical for patients with day-surgery or those presenting with acute coronary syndrome and undergoing urgent PCI. Therefore, fasting SHR may not be applicable for such patients. Then, the retrospective nature of this study introduces unavoidable biases, highlighting the urgent need for a prospective study with a large sample size to provide robust support for our results. Furthermore, it is important to acknowledge that the possibility of selection bias cannot be entirely eliminated as our study only included participants who had simultaneous measurements of FBG and HbA1c levels. Lastly, it should be emphasized that this study solely encompassed the Chinese population, thus necessitating further investigation to ascertain the generalizability of these findings to other populations in different countries.

## Conclusion

In populations undergoing CAG or PCI, both lowest and highest levels of fasting SHR were significantly associated with an increased occurrence of CI-AKI.

## Data availability statement

The original contributions presented in the study are included in the article/**Supplementary Material**. Further inquiries can be directed to the corresponding authors.

## Ethics statement

The studies involving humans were approved by the Ethics Committee of Sir Run Run Shaw Hospital, Zhejiang University (20220228-30). The studies were conducted in accordance with the local legislation and institutional requirements. Written informed consent for participation was not required from the participants or the participants’ legal guardians/next of kin in accordance with the national legislation and institutional requirements.

## Author contributions

YS: Writing – original draft, Writing – review & editing. ML: Conceptualization, Data curation, Writing – original draft. FG: Supervision, Writing – review & editing. SY: Validation, Writing – review & editing. XB: Writing – review & editing. QZ: Writing – review & editing. YT: Writing – review & editing. ZC: Writing – review & editing. DL: Writing – review & editing. WZ: Writing – review & editing. GF: Writing – review & editing. MW: Writing – review & editing.

## References

[1] Piccolo R Giustino G Mehran R Windecker S. Stable coronary artery disease: revascularisation and invasive strategies. Lancet (London England) (2015) 386(9994):702–13. doi: 10.1016/S0140-6736(15)61220-X 26334162

[2] Virani SS Alonso A Aparicio HJ Benjamin EJ Bittencourt MS Callaway CW. Heart disease and stroke statistics-2021 update: A report from the American heart association. Circulation (2021) 143(8):e254–743. doi: 10.1161/CIR.0000000000000950 PMC13036842 33501848

[3] Hakeem A Uretsky BF. Role of postintervention fractional flow reserve to improve procedural and clinical outcomes. Circulation (2019) 139(5):694–706. doi: 10.1161/CIRCULATIONAHA.118.035837 30689413

[4] Mentias A Sarrazin MV Saad M Panaich S Kapadia S Horwitz PA. Long-term outcomes of coronary stenting with and without use of intravascular ultrasound. JACC Cardiovasc Interventions (2020) 13(16):1880–90. doi: 10.1016/j.jcin.2020.04.052 PMC7444477 32819477

[5] Räber L Ueki Y. Outcomes of intravascular ultrasound-guided percutaneous coronary intervention in the United States. JACC Cardiovasc Interventions (2020) 13(16):1891–3. doi: 10.1016/j.jcin.2020.06.031 32819478

[6] Zhao S Chen Y Wang Q Zhu B Wei Z Wang Z. Benefits of successful percutaneous coronary intervention in chronic total occlusion patients with diabetes. Cardiovasc Diabetol (2022) 21(1):271. doi: 10.1186/s12933-022-01708-0 36471410 PMC9724402

[7] Harolds JA. Quality and safety in health care, part XXXIX: complications from the PCI procedure. Clin Nucl Med (2018) 43(9):672–3. doi: 10.1097/RLU.0000000000002025 29742592

[8] James MT Ghali WA Knudtson ML Ravani P Tonelli M Faris P. Associations between acute kidney injury and cardiovascular and renal outcomes after coronary angiography. Circulation (2011) 123(4):409–16. doi: 10.1161/CIRCULATIONAHA.110.970160 21242477

[9] James MT Har BJ Tyrrell BD Faris PD Tan Z Spertus JA. Effect of clinical decision support with audit and feedback on prevention of acute kidney injury in patients undergoing coronary angiography: A randomized clinical trial. Jama (2022) 328(9):839–49. doi: 10.1001/jama.2022.13382 PMC9449791 36066520

[10] McCullough PA Choi JP Feghali GA Schussler JM Stoler RM Vallabahn RC. Contrast-induced acute kidney injury. J Am Coll Cardiol (2016) 68(13):1465–73. doi: 10.1016/j.jacc.2016.05.099 27659469

[11] Li JH He NS. Prevention of iodinated contrast-induced nephropathy. Chin Med J (2011) 124(23):4079–82. 22340345

[12] Stacul F van der Molen AJ Reimer P Webb JA Thomsen HS Morcos SK. Contrast induced nephropathy: updated ESUR Contrast Media Safety Committee guidelines. Eur Radiol (2011) 21(12):2527–41. doi: 10.1007/s00330-011-2225-0 21866433

[13] Seeliger E Sendeski M Rihal CS Persson PB. Contrast-induced kidney injury: mechanisms, risk factors, and prevention. Eur Heart J (2012) 33(16):2007–15. doi: 10.1093/eurheartj/ehr494 22267241

[14] Stolker JM McCullough PA Rao S Inzucchi SE Spertus JA Maddox TM. Pre-procedural glucose levels and the risk for contrast-induced acute kidney injury in patients undergoing coronary angiography. J Am Coll Cardiol (2010) 55(14):1433–40. doi: 10.1016/j.jacc.2009.09.072 20359592

[15] Yuan Y Qiu H Hu XY Luo T Gao XJ Zhao XY. Risk factors of contrast-induced acute kidney injury in patients undergoing emergency percutaneous coronary intervention. Chin Med J (2017) 130(1):45–50. doi: 10.4103/0366-6999.196578 28051022 PMC5221111

[16] Roberts GW Quinn SJ Valentine N Alhawassi T O'Dea H Stranks SN. Relative hyperglycemia, a marker of critical illness: introducing the stress hyperglycemia ratio. J Clin Endocrinol Metab (2015) 100(12):4490–7. doi: 10.1210/jc.2015-2660 26485219

[17] Chu H Huang C Tang Y Dong Q Guo Q. The stress hyperglycemia ratio predicts early hematoma expansion and poor outcomes in patients with spontaneous intracerebral hemorrhage. Ther Adv Neurological Disord (2022) 15:17562864211070681. doi: 10.1177/17562864211070681 PMC8785298 35082921

[18] Huang YW An YH Yin XS Li ZP. Association of the stress hyperglycemia ratio and clinical outcomes in patients with cardiovascular diseases: a systematic review and meta-analysis. Eur Rev Med Pharmacol Sci (2022) 26(24):9258–69. doi: 10.26355/eurrev_202212_30679 36591838

[19] Liu B Chen Y Yu L Zhou M. Stress hyperglycemia ratio is associated with systemic inflammation and clinical outcomes in diabetic inpatients with pneumonia on admission. J Diab (2023) 15(7):545–56. doi: 10.1111/1753-0407.13398 PMC10345973 37144245

[20] Zhang Y Song H Bai J Xiu J Wu G Zhang L. Association between the stress hyperglycemia ratio and severity of coronary artery disease under different glucose metabolic states. Cardiovasc Diabetol (2023) 22(1):29. doi: 10.1186/s12933-023-01759-x 36755256 PMC9909934

[21] Cui K Fu R Yang J Xu H Yin D Song W. The impact of fasting stress hyperglycemia ratio, fasting plasma glucose and hemoglobin A1c on in-hospital mortality in patients with and without diabetes: findings from the China acute myocardial infarction registry. Cardiovasc Diabetol (2023) 22(1):165. doi: 10.1186/s12933-023-01868-7 37403082 PMC10320917

[22] Fu R Cui K Yang J Xu H Yin D Song W. Fasting stress hyperglycemia ratio and in-hospital mortality after acute myocardial infarction in patients with different glucose metabolism status: Results from China acute myocardial infarction registry. Diabetes Res Clin Practice (2023) 196:110241. doi: 10.1016/j.diabres.2023.110241 36623641

[23] Nathan DM Kuenen J Borg R Zheng H Schoenfeld D Heine RJ. Translating the A1C assay into estimated average glucose values. Diabetes Care (2008) 31(8):1473–8. doi: 10.2337/dc08-0545 PMC2742903 18540046

[24] Silver SA Shah PM Chertow GM Harel S Wald R Harel Z. Risk prediction models for contrast induced nephropathy: systematic review. BMJ (Clinical Res ed) (2015) 351:h4395. doi: 10.1136/bmj.h4395 PMC4784870 26316642

[25] Nusca A Lauria Pantano A Melfi R Proscia C Maddaloni E Contuzzi R. Glycemic variability assessed by continuous glucose monitoring and short-term outcome in diabetic patients undergoing percutaneous coronary intervention: an observational pilot study. J Diabetes Res (2015) 2015:250201. doi: 10.1155/2015/250201 26273664 PMC4529948

[26] Xia J Xu J Hu S Hao H Yin C Xu D. Impact of glycemic variability on the occurrence of periprocedural myocardial infarction and major adverse cardiovascular events (MACE) after coronary intervention in patients with stable angina pectoris at 6months follow-up. Clin Chim Acta; Int J Clin Chem (2017) 471:196–200. doi: 10.1016/j.cca.2017.06.014 28624498

[27] Xu W Yang YM Zhu J Wu S Wang J Zhang H. Predictive value of the stress hyperglycemia ratio in patients with acute ST-segment elevation myocardial infarction: insights from a multi-center observational study. Cardiovasc Diabetol (2022) 21(1):48. doi: 10.1186/s12933-022-01479-8 35351149 PMC8962934

[28] Mi D Li Z Gu H Jiang Y Zhao X Wang Y. Stress hyperglycemia is associated with in-hospital mortality in patients with diabetes and acute ischemic stroke. CNS Neurosci Ther (2022) 28(3):372–81. doi: 10.1111/cns.13764 PMC8841306 35084107

[29] Wei QC Chen YW Gao QY Ren KD Liu YB He F. Association of stress hyperglycemia with clinical outcomes in patients with ST-elevation myocardial infarction undergoing percutaneous coronary intervention: a cohort study. Cardiovasc Diabetol (2023) 22(1):85. doi: 10.1186/s12933-023-01812-9 37046267 PMC10100063

[30] Yang J Zheng Y Li C Gao J Meng X Zhang K. The impact of the stress hyperglycemia ratio on short-term and long-term poor prognosis in patients with acute coronary syndrome: insight from a large cohort study in Asia. Diabetes Care (2022) 45(4):947–56. doi: 10.2337/dc21-1526 35045167

[31] Yang Y Kim TH Yoon KH Chung WS Ahn Y Jeong MH. The stress hyperglycemia ratio, an index of relative hyperglycemia, as a predictor of clinical outcomes after percutaneous coronary intervention. Int J Cardiol (2017) 241:57–63. doi: 10.1016/j.ijcard.2017.02.065 28256326

[32] Abdu FA Galip J Qi P Zhang W Mohammed AQ Liu L. Association of stress hyperglycemia ratio and poor long-term prognosis in patients with myocardial infarction with non-obstructive coronary arteries. Cardiovasc Diabetol (2023) 22(1):11. doi: 10.1186/s12933-023-01742-6 36647062 PMC9843969

[33] Zhou Y Liu L Huang H Li N He J Yao H. 'Stress hyperglycemia ratio and in-hospital prognosis in non-surgical patients with heart failure and type 2 diabetes. Cardiovasc Diabetol (2022) 21(1):290. doi: 10.1186/s12933-022-01728-w 36572923 PMC9791974

[34] Couto SMF da Fonseca CD Watanabe M de Fátima Fernandes Vattimo M. Protection of coenzyme Q10 against contrast-induced acute kidney injury in male diabetic rats. Diabetol Metab Syndrome (2021) 13(1):69. doi: 10.1186/s13098-021-00689-6 PMC8207798 34134745

[35] DeFronzo RA Reeves WB Awad AS. Pathophysiology of diabetic kidney disease: impact of SGLT2 inhibitors. Nat Rev Nephrol (2021) 17(5):319–34. doi: 10.1038/s41581-021-00393-8 33547417

[36] Jha JC Ho F Dan C Jandeleit-Dahm K. A causal link between oxidative stress and inflammation in cardiovascular and renal complications of diabetes. Clin Sci (London Engl 1979) (2018) 132(16):1811–36. doi: 10.1042/CS20171459 30166499

[37] Paneni F Beckman JA Creager MA Cosentino F. Diabetes and vascular disease: pathophysiology, clinical consequences, and medical therapy: part I. Eur Heart J (2013) 34(31):2436–43. doi: 10.1093/eurheartj/eht149 PMC3743069 23641007

[38] Peng J Li X Zhang D Chen JK Su Y Smith SB. Hyperglycemia, p53, and mitochondrial pathway of apoptosis are involved in the susceptibility of diabetic models to ischemic acute kidney injury. Kidney Int (2015) 87(1):137–50. doi: 10.1038/ki.2014.226 PMC4276728 24963915

[39] Xu L Zhou Y Wang G Bo L Jin B Dai L. The UDPase ENTPD5 regulates ER stress-associated renal injury by mediating protein N-glycosylation. Cell Death Disease (2023) 14(2):166. doi: 10.1038/s41419-023-05685-4 36849424 PMC9971188

[40] Kanwar YS Sun L Xie P Liu FY Chen S. A glimpse of various pathogenetic mechanisms of diabetic nephropathy. Annu Rev Pathol (2011) 6:395–423. doi: 10.1146/annurev.pathol.4.110807.092150 21261520 PMC3700379

[41] Lee TF Tommasi S Bersten A Heilbronn LK Sotgia S Zinellu A. Does hyperglycemia affect arginine metabolites in critically ill patients? A prospective cohort and in *vitro* study. Diabetol Metab Syndrome (2023) 15(1):68. doi: 10.1186/s13098-023-01035-8 PMC10067243 37005603

[42] Prabhakar S Starnes J Shi S Lonis B Tran R. Diabetic nephropathy is associated with oxidative stress and decreased renal nitric oxide production. J Am Soc Nephrol JASN (2007) 18(11):2945–52. doi: 10.1681/ASN.2006080895 17928507

[43] Busch M Franke S Rüster C Wolf G. Advanced glycation end-products and the kidney. Eur J Clin Invest (2010) 40(8):742–55. doi: 10.1111/j.1365-2362.2010.02317.x 20649640

[44] Tuleta I Frangogiannis NG. Diabetic fibrosis. Biochim Biophys Acta Mol basis Disease (2021) 1867(4):166044. doi: 10.1016/j.bbadis.2020.166044 PMC7867637 33378699

[45] Connelly KA Yan AT Leiter LA Bhatt DL Verma S. Cardiovascular implications of hypoglycemia in diabetes mellitus. Circulation (2015) 132(24):2345–50. doi: 10.1161/CIRCULATIONAHA.115.015946 26667098

[46] Hanefeld M Duetting E Bramlage P. Cardiac implications of hypoglycaemia in patients with diabetes - a systematic review. Cardiovasc Diabetol (2013) 12:135. doi: 10.1186/1475-2840-12-135 24053606 PMC3849493

[47] Lee YL Chen BK Lin KD Su RW Lee MY Hsiao PJ. The impact of severe hypoglycemia on renal impairment in type 2 diabetes. Diabetes Res Clin Practice (2015) 108(3):448–55. doi: 10.1016/j.diabres.2015.02.028 25779866

[48] Cavalot F. Do data in the literature indicate that glycaemic variability is a clinical problem? Glycaemic variability and vascular complications of diabetes. Diab Obes Metab (2013) 15(Suppl 2):3–8. doi: 10.1111/dom.12140 24034513

[49] Gorst C Kwok CS Aslam S Buchan I Kontopantelis E Myint PK. Long-term glycemic variability and risk of adverse outcomes: A systematic review and meta-analysis. Diabetes Care (2015) 38(12):2354–69. doi: 10.2337/dc15-1188 26604281

