# Supplementary material for: Association between fasting stress hyperglycemia ratio and contrast-induced acute kidney injury in coronary angiography patients: a cross-sectional study
Source: Front Endocrinol (Lausanne). 2023 Dec 13;14:1300373. doi: 10.3389/fendo.2023.1300373 (PMC10753820; doi:10.3389/fendo.2023.1300373)

**Supplementary material**

**Supplementary Table 1. Modified Poisson’s regression analysis of fasting SHR categories with CI-AKI in a sensitivity analysis by excluding patients with hyperglycemic crises at baseline.**

**P2**

**Supplementary Table 2.** **Modified Poisson’s regression analysis of fasting SHR categories with CI-AKI in a sensitivity analysis by excluding patients with hypoglycemia at baseline.**

**P3**

**Supplementary Figure 1. Association between fasting SHR and CI-AKI in two sensitivity analyses by RCS model.**

**P4**

**Supplementary Table 1** Modified Poisson’s regression analysis of fasting SHR categories with CI-AKI in a sensitivity analysis by excluding patients with hyperglycemic crises at baseline.

| SHR | Cases/Overall (%) | Model 1 |  |  | Model 2 |  |  | Model 3 |  |
| --- | --- | --- | --- | --- | --- | --- | --- | --- | --- |
|  |  | aRR (95%CI) | *P* value |  | aRR (95%CI) | *P* value |  | aRR (95%CI) | *P* value |
| < 0.7 | 93/537 (17.3%) | 1.857 [1.389 to 2.482] | < 0.001* |  | 1.830 [1.355 to 2.471] | < 0.001* |  | 1.796 [1.317 to 2.449] | < 0.001* |
| ≤ 0.7, < 0.9 | 125/1233 (10.1%) | 1 (Reference) |  |  | 1 (Reference) |  |  | 1 (Reference) |  |
| ≤ 0.9, < 1.1 | 113/719 (15.7%) | 1.653 [1.258 to 2.172] | < 0.001* |  | 1.611 [1.218 to 2.132] | 0.001* |  | 1.629 [1.223 to 2.171] | 0.001* |
| ≤ 1.1, < 1.3 | 57/300 (19.0%) | 2.079 [1.476 to 2.929] | < 0.001* |  | 2.079 [1.458 to 2.965] | < 0.001* |  | 2.090 [1.451 to 3.011] | < 0.001* |
| ≥ 1.3 | 72/292 (24.7%) | 2.901 [2.098 to 4.011] | < 0.001* |  | 2.741 [1.961 to 3.831] | < 0.001* |  | 2.550 [1.805 to 3.603] | < 0.001* |
| *P* for trend |  |  | < 0.001* |  |  | < 0.001* |  |  | < 0.001* |

Model 1: Adjusted for none.

Model 2: Adjusted for age, gender (male or female), hypertension (yes or no), eGFR, type of contrast agent (isotonic or hypotonic), volume of contrast, and LVEF.

Model 3: Additionally adjusted for CRP (<5, 5-10, or ≥10mg/L), and medications (administration of statin, furosemide injection and dopamine) (yes or no).

SHR: stress hyperglycemia ratio; CI-AKI: contrast-induced acute kidney injury; aRR: adjusted relative risk; CI: confidence interval; eGFR: estimated glomerular filtration rate; LVEF: left ventricular ejection fraction; CRP: C-reactive protein.

**Supplementary Table 2** Modified Poisson’s regression analysis of fasting SHR categories with CI-AKI in a sensitivity analysis by excluding patients with hypoglycemia at baseline.

| SHR | Cases/Overall (%) | Model 1 |  |  | Model 2 |  |  | Model 3 |  |
| --- | --- | --- | --- | --- | --- | --- | --- | --- | --- |
|  |  | aRR (95%CI) | *P* value |  | aRR (95%CI) | *P* value |  | aRR (95%CI) | *P* value |
| < 0.7 | 82/503 (16.3%) | 1.750 [1.294 to 2.366] | < 0.001* |  | 1.753 [1.283 to 2.394] | < 0.001* |  | 1.758 [1.273 to 2.427] | 0.001* |
| ≤ 0.7, < 0.9 | 122/1218 (10.0%) | 1 (Reference) |  |  | 1 (Reference) |  |  | 1 (Reference) |  |
| ≤ 0.9, < 1.1 | 112/714 (15.7%) | 1.671 [1.269 to 2.201] | < 0.001* |  | 1.611 [1.215 to 2.137] | 0.001* |  | 1.628 [1.219 to 2.174] | 0.001* |
| ≤ 1.1, < 1.3 | 60/306 (19.6%) | 2.191 [1.562 to 3.074] | < 0.001* |  | 2.193 [1.543 to 3.116] | < 0.001* |  | 2.150 [1.497 to 3.088] | < 0.001* |
| ≥ 1.3 | 86/313 (27.5%) | 3.403 [2.494 to 4.644] | < 0.001* |  | 3.176 [2.301 to 4.385] | < 0.001* |  | 2.923 [2.093 to 4.080] | < 0.001* |
| *P* for trend |  |  | < 0.001* |  |  | < 0.001* |  |  | < 0.001* |

Model 1: Adjusted for none.

Model 2: Adjusted for age, gender (male or female), hypertension (yes or no), eGFR, type of contrast agent (isotonic or hypotonic), volume of contrast, and LVEF.

Model 3: Additionally adjusted for CRP (<5, 5-10, or ≥10mg/L), and medications (administration of statin, furosemide injection and dopamine) (yes or no).

SHR: stress hyperglycemia ratio; CI-AKI: contrast-induced acute kidney injury; aRR: adjusted relative risk; CI: confidence interval; eGFR: estimated glomerular filtration rate; LVEF: left ventricular ejection fraction; CRP: C-reactive protein.

**Supplementary Figure 1.** **Association between fasting SHR and CI-AKI in two sensitivity analyses by RCS model.** (A) a sensitivity analysis by excluding patients with hypoglycemia at baseline; (B) a sensitivity analysis by excluding patients with hyperglycemic crises at baseline; The relative risk of fasting SHR for CI-AKI is represented by the solid blue line, while the shaded area surrounding the line represents the 95% CI of the curve. CI-AKI: contrast-induced acute kidney injury; CI: confidence interval; RCS: restricted cubic spline; SHR: stress hyperglycemia ratio.


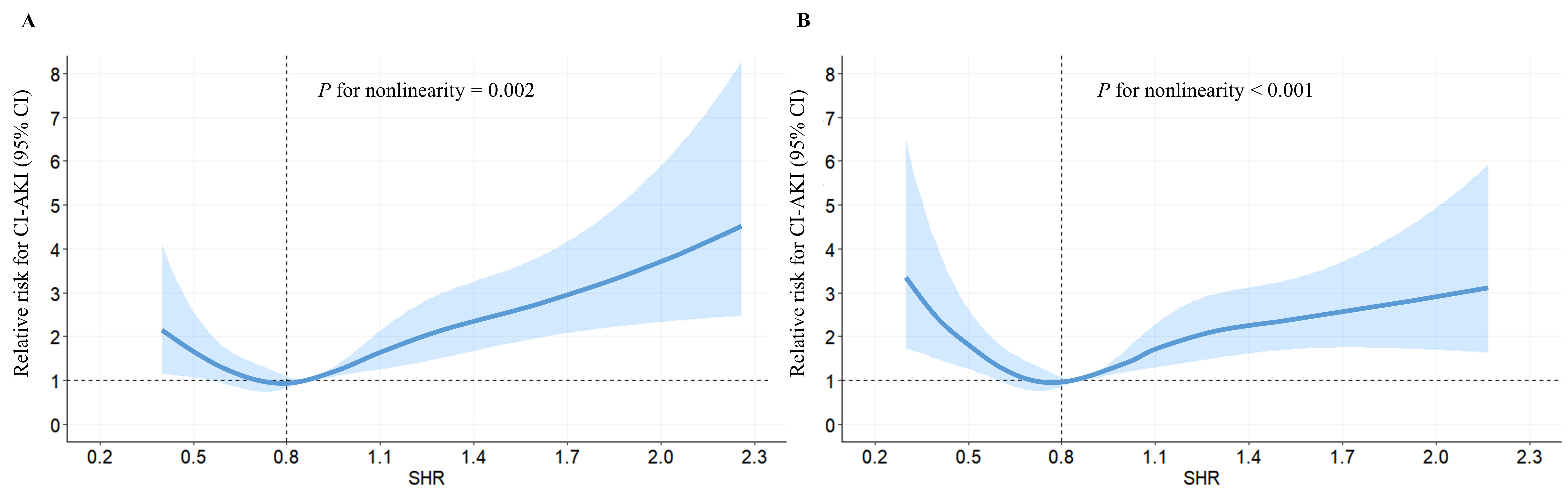

Supplement: Supplementary file 1 [file DataSheet_1.docx]
